# Effect of breathing patterns on mandibular cortical bone quality in children and establishment of a preliminary screening model

**DOI:** 10.1186/s12903-023-03406-z

**Published:** 2023-10-27

**Authors:** Gaoli Wang, Badr Sultan Saif, Bo Cheng, Hongfei Li, Yutong Li, Jiawen Liu, Xiaoyong Ren, Rui Zou, Fei Wang

**Affiliations:** 1 Key Laboratory of Shaanxi Province for Craniofacial Precision Medicine Research, Clinical Research Center of Shaanxi Province for Dental and Maxillofacial Diseases, College of Stomatology, Xi’an Jiaotong University, Xi’an, Shaanxi 710004 People’s Republic of China; 2 Department of Orthodontics, Xi’an Jiaotong University, Xi’an, China; 3 Department of Orthodontics, The First Affiliated Hospital of Xi’an Jiaotong University, Xi’an, China; 4 The Fourth Outpatient Department, Xi’An Jiaotong University Stomatological Hospital, Xi’an, China; 5 Department of Otorhinolaryngology-Head and Neck Surgery, The Second Affiliated Hospital of Xi’an Jiaotong University, Xi’an, China

**Keywords:** Breathing patterns, Bone quality, CBCT

## Abstract

**Objective:**

This retrospective study analyzed breathing patterns and age subgroups effect on cortical bone quality of the mandible in growing subjects, aiming to explore the application value of facial skeletal pattern combined with cortical bone density detection in early screening diagnosis of mouth breathing.

**Methods:**

One hundred twenty-six participants were divided into four groups: mouth breathing group (7–9, 10–12 years old) and nasal breathing group (7–9, 10–12 years old). The mandibular anterior, middle, and posterior cortical bone mineral density (CBMD), cortical bone width (MCW), ANB, and FMA values were measured. Independent T-test and Mann–Whitney U test were used to compare the measured values. Binary logistic regression was employed to analyze the correlation between measured variables and the children’s breathing patterns. ROC analysis was used to determine the ability of the cortical bone density measurements in early screening diagnosis of MB.

**Results:**

Mouth breathing had a negative impact on CBMD and MCW of the pre-mandibular (Pog) in subjects aged 7–9 years and also impacted the development of (Pog) and submandibular (Me) sites in subjects aged 10–12 years. Older children in the nasal breathing group have higher CBMD, MCW, and SNB values and lower FMA values. Single-factor and multiple-factor logistic binary regression analysis showed that FMA, MSPogCBMD, MSPogMCW, and ANB are correlated factors for children at risk of mouth breathing.

**Conclusion:**

Mouth breathing pattern is closely associated with decreased mandibular CBMD and MCW values in children aged 7–12, where the anterior (Pog) and inferior (Me) sites of anterior mandible are more significantly affected. Furthermore, in combination with facial skeletal pattern, it provides a basis for the early warning diagnosis of mouth breathing.

## Background

The growth of the craniofacial region involves significant changes in the facial dimension. Among the craniofacial structures, the mandible plays a crucial role in maintaining proper facial appearance and function, and any abnormalities in mandibular development can lead to various dental and functional problems, such as malocclusion, sleep apnea, and facial asymmetry [1, 2]. Therefore, understanding the factors influencing mandibular development is essential for preventing and treating these conditions.

Partial obstruction of the airways frequently due to chronic adenoid hypertrophy leads to mouth breathing and altered oral muscle activity, resulting in the posterior rotation of the mandible, causing an increased overjet. Simultaneously, this restricted mandibular rotation can contribute to a skeletal open bite [3, 4]. Conversely, Class III malocclusion may arise in response to tonsillar hypertrophy, which forces the mandible forward to widen the oropharyngeal airway, creating an anterior crossbite [5, 6]. Both malocclusions and the associated skeletal open bite can culminate in facies adenoidea, characterized by distinct facial features like a long face and open mouth posture, representing adaptive responses to chronic airway obstruction during growth. Therefore, muscular activity imbalance caused by mouth breathing can increase the negative pressure in the oral cavity, which may lead to changes in the position, shape, and size of the mandible.

A study performed by Eimar et al. investigated the mandibular bone quality in children using panoramic radiographs and reported that the altered normal breathing was associated with significantly reduced mandibular cortical width compared to typically nasal breathing individuals [7]. Another study that investigated the same area included subjects with obstructive sleep apnea (OSA) and compared them to healthy controls. The results of this study revealed that children with OSA had significantly lower mandibular bone density and altered mandibular morphology compared to the healthy controls [8]. These findings suggest that mouth breathing patterns may negatively influence mandibular bone quality in children. The effect of breathing patterns on children’s mandibular bone quality is still not fully understood. However, it is essential to note that more research is needed to fully understand the underlying mechanisms and establish a cause-and-effect relationship. The studies conducted thus far are limited in size and scope, and further research is needed to confirm the findings where breathing patterns may affect mandibular bone quality.

Fractal dimension analysis offers a mathematical approach for assessing the complexity and irregularity of bone structures. A radiation-free alternative to objectively quantifying bone density reported by Jurczyszyn et al. [9], which can contribute to a more comprehensive understanding of OSA-related factors for potentially examining the relationships between OSA severity and bone health. Dental CBCT has been widely used for mouth implantation and orthodontic diagnosis and treatment planning; besides, it has the advantages of low radiation dose and high spatial resolution. It can also be employed to evaluate the CBMD and MCW of the mandible before the implantation of micro implant screws. It provides a precise depiction of a scanned human skull and possesses significant clinical utility [10]. Berco et al. reported that CBCT could be used for three-dimensional linear measurement of the craniofacial complex, which has proven to be clinically accurate and reliable [11].

Therefore, we aim to evaluate the cortical bone mass of the mandible in children aged 7–12 with different breathing patterns using CBCT data.

## Methods

### Ethical approval

The Stomatological Hospital of Xi’an Jiaotong University Ethics Committee has approved this study. Ethical approval number: Xjkqll [2018] No.17.

### Sample size calculation

Using a formula proposed by Pandis [12], the sample size was calculated with power = 80%, significance level = 0.05 to detect a difference of 0.5 mm in cortical bone width between MB and NB, and standard deviation = 0.7 mm [7]. It was found that thirty subjects per subgroup would be adequate.

### Subjects

**Fig. 1:**
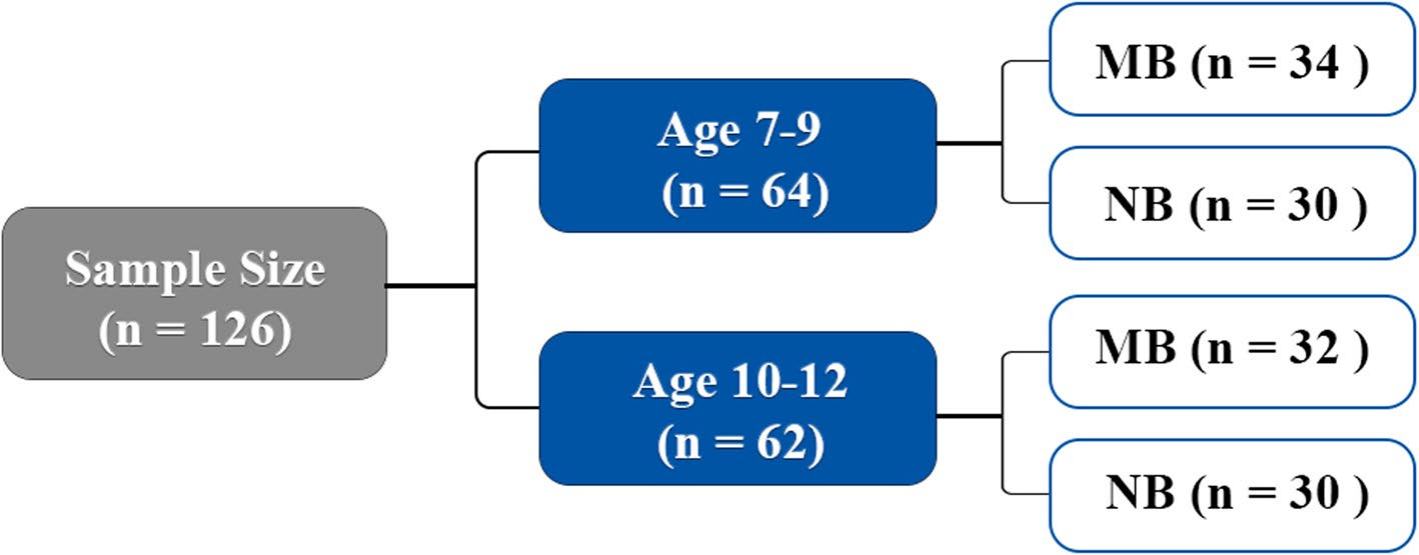
Sample size

In this investigation, 126 CBCT scans were gathered from 126 individuals (61 males and 65 females) (Fig. 1). These CBCT scans were acquired as diagnostic documentation of children aged 7–12 years who first attended the Orthodontics Center at the Stomatological Hospital of Xi’an Jiaotong University from 2018 to 2023. Exclusion criteria include (a) a History of orthodontic or orthognathic surgery, (b) Severe craniofacial asymmetry, (c) Children with known hereditary or systemic medical conditions that substantially affect the metabolism of bone or those who consume medicines proven to affect bone metabolism. Based on the medical history, any patients who had previously undergone orthodontic treatment or had any syndromes in the head and neck area were excluded from the investigation. Participants’ guardians have provided their informed and written consent prior to diagnostic CBCT scanning and participation in the study.

### CT process

Each participant was given directions to be positioned correctly, sit upright, keep optimum intercuspation of their jaws, and verify that the lips and tongue were relaxed. The patients’ Frankfort horizontal plane was kept parallel to the floor, and they were instructed to breathe properly through the nose, keeping their head and tongue stable. CBCT scans were captured utilizing (i-Cat, Imaging Sciences International, Hatfield, PA, USA) cone beam machine at 120 kV, 5 mA, 14 × 17 cm FOV, 0.4 mm voxel, with a scan duration of 8.9 s. The CBCT images were then saved in DICOM format (digital imaging and communications in medicine).

### Breathing pattern diagnostic criteria

A multidisciplinary team consisting of an orthodontist and an ENT specialist evaluated the diagnostic criteria for respiratory patterns. First, the orthodontist screens for mouth respiration, which includes (1) Asking parents to record a video, checking whether the child’s lips are closed after falling asleep, whether snoring can be heard, and whether there are symptoms of drooling on the pillow, (2) Asking if the child is prone to fatigue, allergies, and colds, (3) Clinical examination of the child’s habitual lip posture, nostril shape, and size and perform Glatzel mirror test [13]. All subjects underwent an otolaryngologist’s examination, including nasopharyngeal X-ray, nasal endoscopy, and comprehensive nasopharyngoscopy, and were diagnosed as mouth breathing based on the presence or absence of nasopharyngeal airway obstruction [14].

### CBCT orientation and measurements

**Fig. 2:**
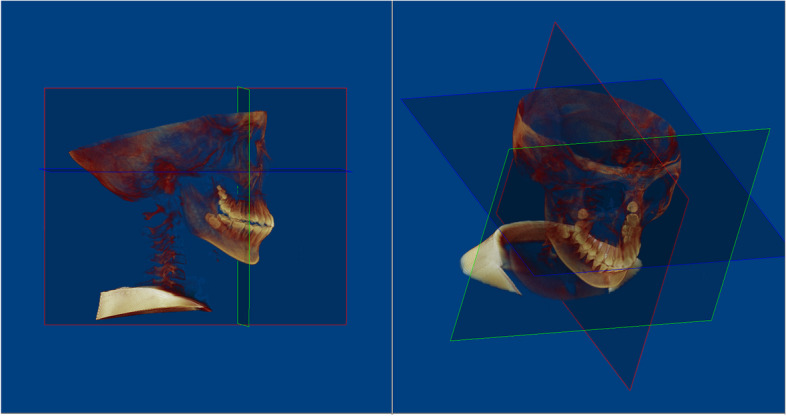
Orientation and alignment of CBCT with reference planes

The DICOM files were imported for measurements using Dolphin Imaging software (Dolphin Imaging & Management Solutions®, Chatsworth, CA, USA) version 11.7. The axial plane of the CBCT images had been aligned with the Frankfort horizontal plane (FHP), the midsagittal plane was matched with the patient’s midline at Nasion (N) point, and the coronal plane had been modified to be perpendicular to the axial plane and passing through Porion point (Fig. 2). One investigator who was unaware of the participants’ demographics recorded the measurements.

**Fig. 3:**
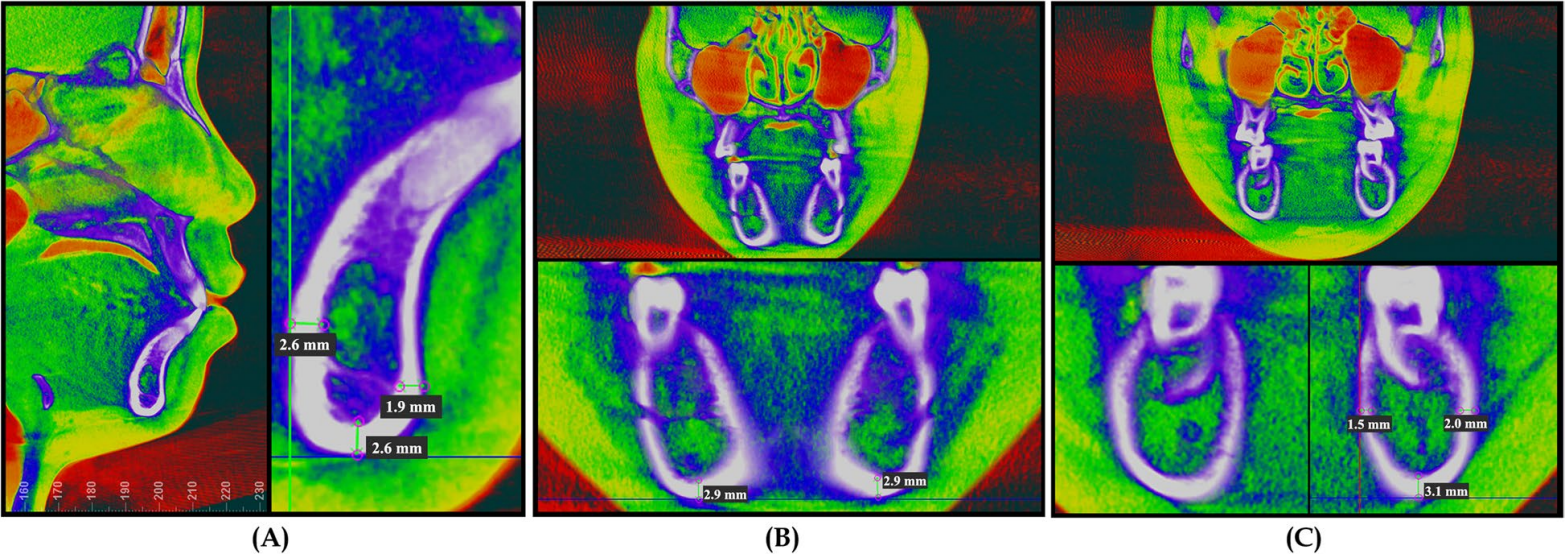
Areas Selected for measurements, **A** Mandibular symphysis, **B** Middle of the mandible, **C** Posterior of the mandible

In the initially aligned CBCT images, the cross sections of the anterior, middle, and posterior segments of each subject’s mandible were selected through the following steps: 1. Mandibular symphysis, chosen at the Sagittal plane section images of the anterior mandible (Fig. 3A); 2. Middle of the mandible: Coronal section images perpendicular to the median sagittal plane and parallel to the bilateral mental foramen (Fig. 3B); 3. Posterior of the mandible: A coronal image was selected perpendicular to the median sagittal plane and parallel to the root bifurcation of the bilateral first molars (Fig. 3C).

**Fig. 4:**
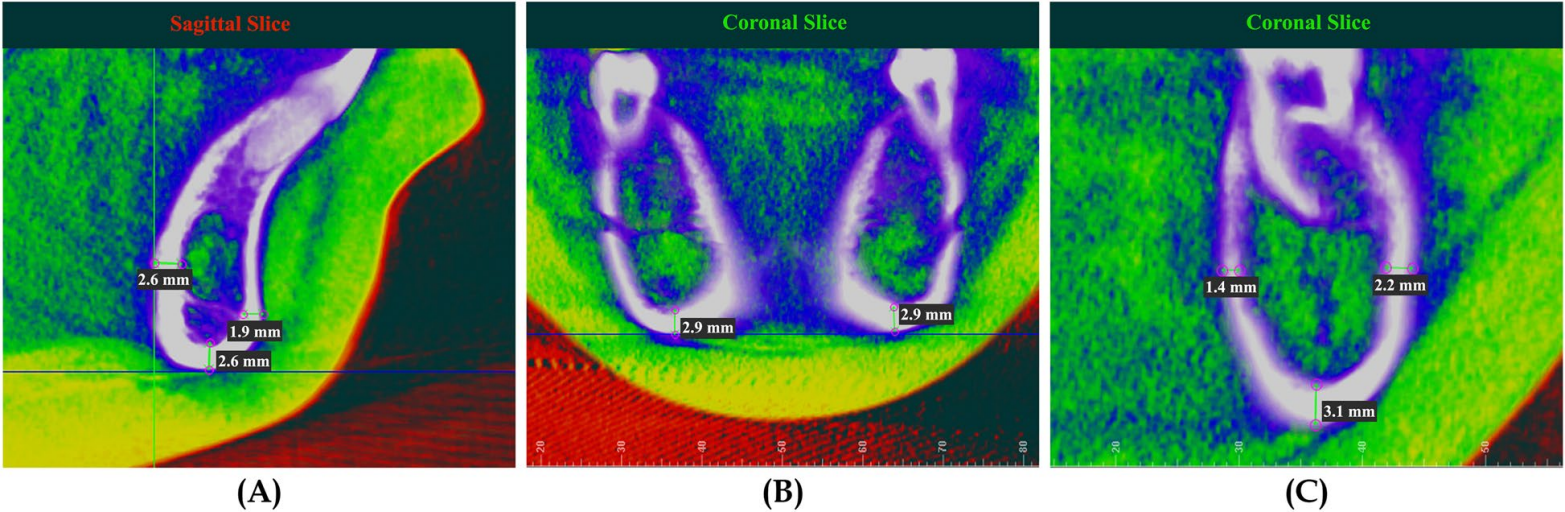
Specific areas selected for MCW measurements

**Fig. 5:**
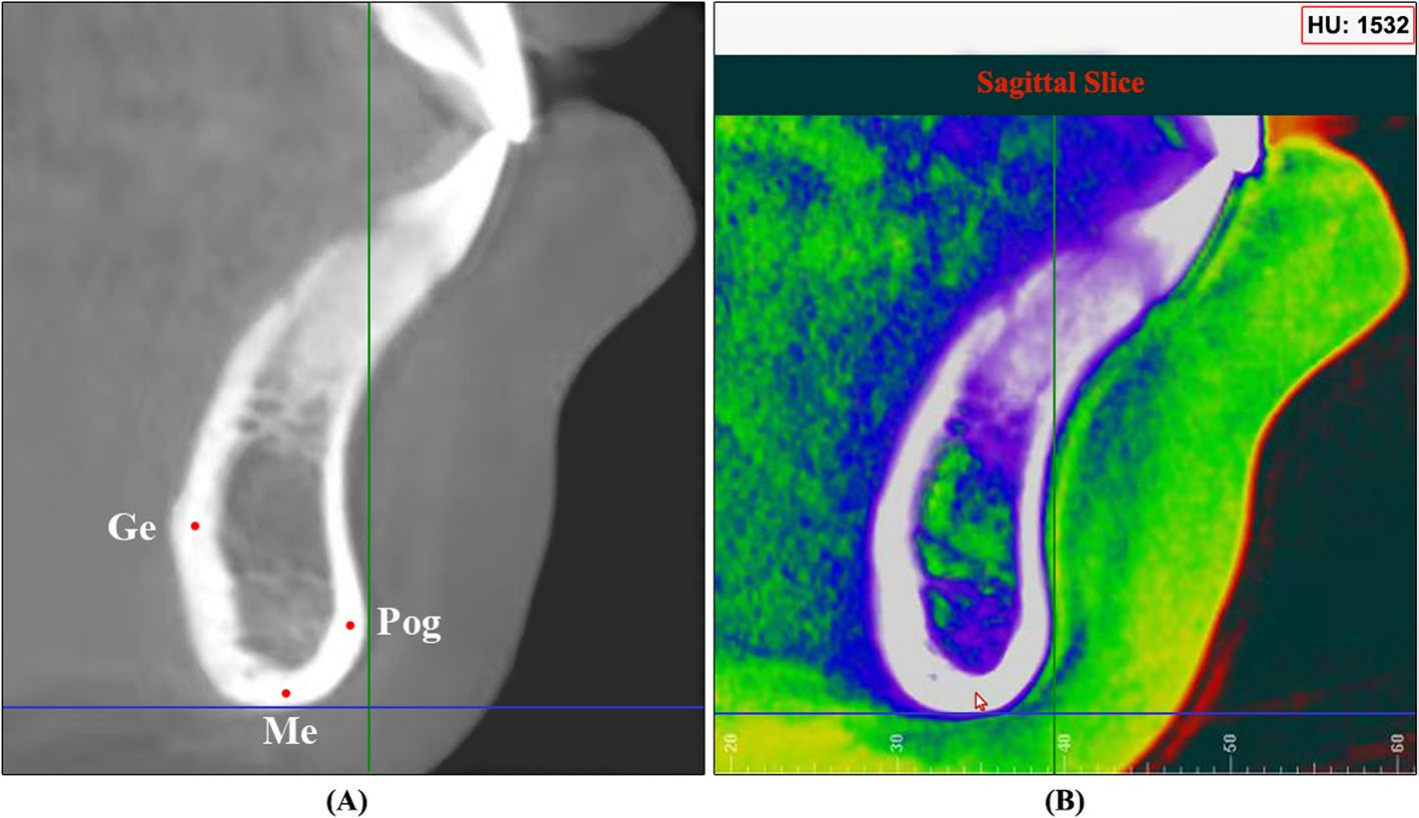
Measurement of CBMD. **A** Sites for calculation of CBMD in anterior mandible, **B** Movement of the cursor over selected site to show HU value

MCW values were measured as the linear distance at each planned selected area. For mandibular symphysis (MS), the MCW was measured at pogonion (Pog), Menton (Me), and genial tubercle (Ge) areas. For the middle of the mandible, the MCW values were measured at one location, which is inferior or basal cortical bone width (IMF). While for the posterior mandible, MCW values were measured at buccal (B6), lingual (L6), and inferior areas (I6) (Table 1). All MCW measurements for the middle and posterior mandible were taken for both the right and left sides (Fig. 4A, B, and C). For CBMD values, the location of the points selected by moving the cursor to the middle of each previously selected location used for MCW measurements. Dolphin software then provides Hounsfield Units (HU) bone density values for each point selected (Fig. 5A and B). Steiner’s analysis has been used for the cephalometric measurements.

**Table 1:** Abbreviation of the landmarks

Abbreviation	Definition
RP	Respiratory pattern
MS	mandibular symphysis
Pog	Pogonion
Me	Mention
Ge	Posterior point of the symphysis in the area of the genial tubercles
IMF	Inferior to the mental foramen
L6	Lingual side of the first molar
B6	Buccal side of the first molar
I6	Inferior to the First molar

### Statistical analysis

All measurements were analyzed using SPSS software (version 27.0, Chicago, IL). The Shapiro–Wilk test was used to test the normality of the variables. Twenty CBCT images were randomly selected to investigate the reliability of the measurement method used. The two researchers repeated all the measurements one week after the first attempt. Inter-investigator and intra-investigator errors were evaluated using intra-group correlation coefficient tests to calculate measurement errors.

The 126 participants were grouped according to age and breathing patterns. Mandibular CBMD and MCW values conforming to normal distribution were expressed as mean ± standard deviation (± s), and independent sample t-test was used. Parameters that did not conform to the normal distribution were represented by the median (quartile distance) (M(IQR)). The Mann–Whitney test was used to compare the differences in CBMD and CWM among different breathing patterns and age groups. Mouth respiration (binary outcome index) was used as the dependent variable; age, ANB, SNA, SNB, FMA, CBMD, and MCW at each site were used as covariates for single-factor screening. The logistic regression model was established by “entry” method, and the screening criteria were α_in = 0.05 and α_out = 0.10. Sites with statistically significant results were selected as predictors by binary logistic regression (no multicollinearity between factors, VIF < 10). The regression equation is established. Receiver operating characteristic curve (ROC) was plotted.

## Results

**Fig. 6:**
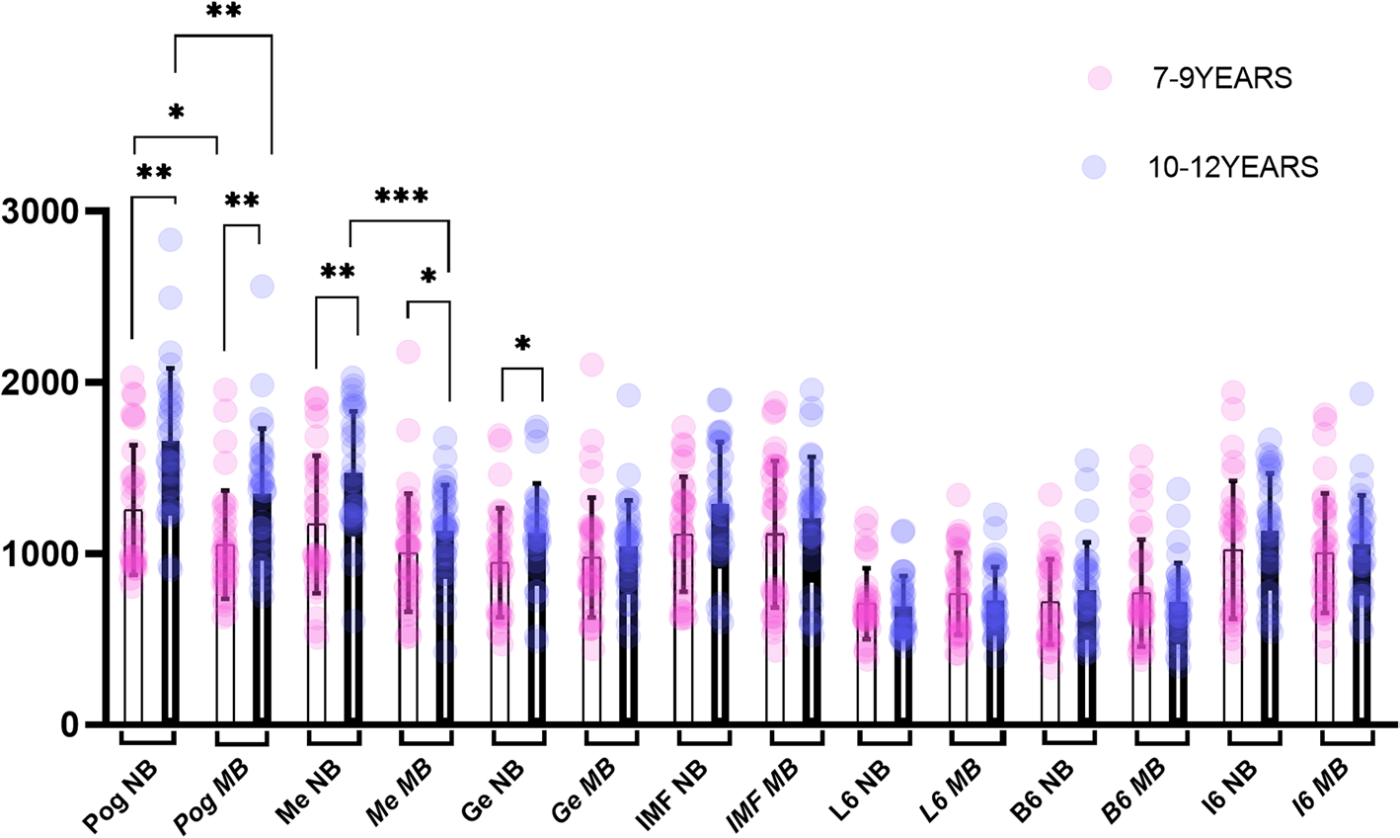
CBMD comparison between mouth and nasal breathing groups in children aged 7–9 and 10–12

**Fig. 7:**
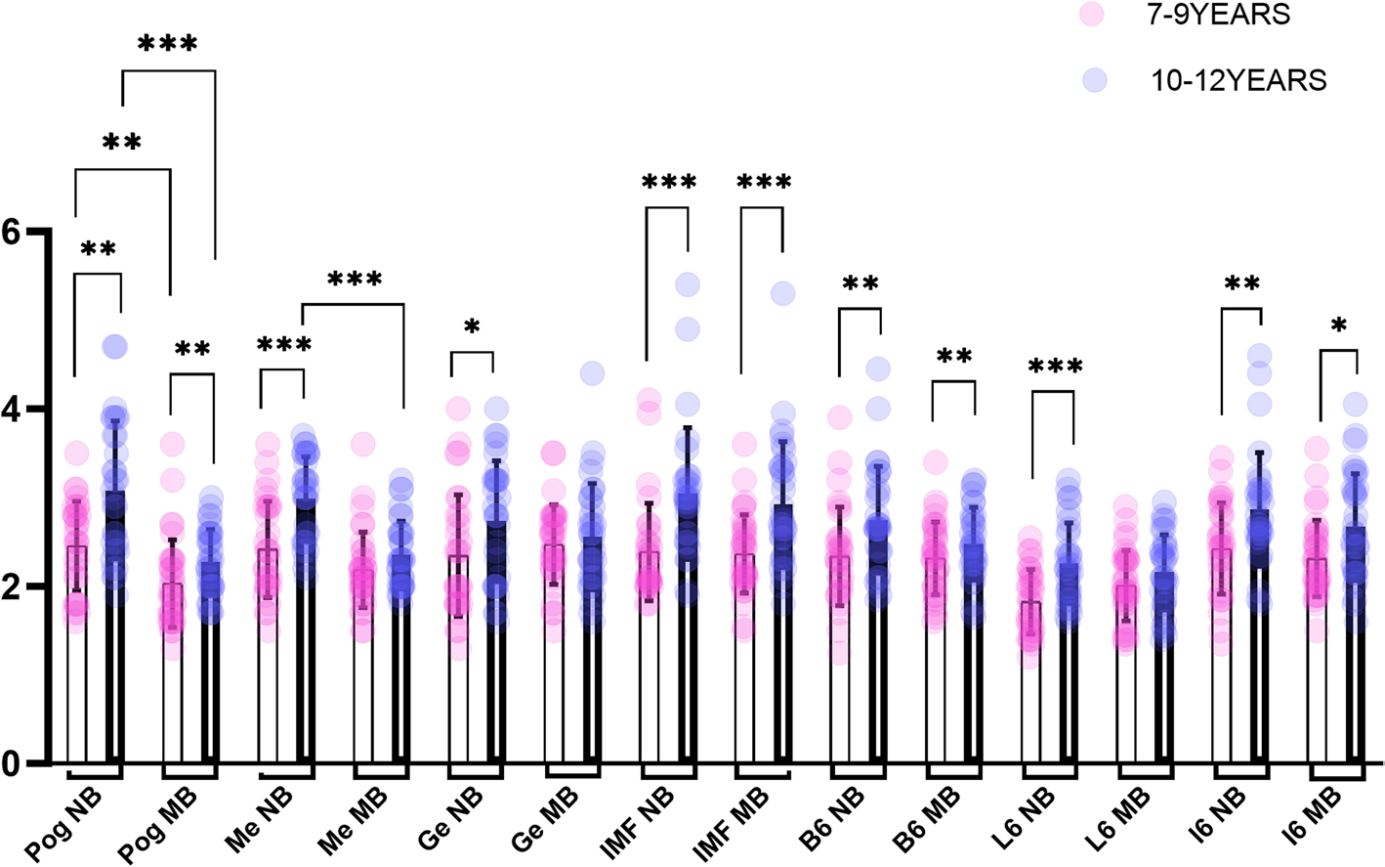
MCW comparison between mouth and nasal breathing groups in children aged 7–9 and 10–12

The results of the (ICC) test showed consistency in measurements within the same researcher (0.90—0.95) and between researchers (0.89—0.95), indicating good reliability of the measurement method used. The Shapiro–Wilk test results indicated that the (MSMeCBMD, I6CBMD, and L6MCW) values were normally distributed where independent sample t-test was used to determine the significant difference. In contrast, the Mann–Whitney U test was used for the remaining sites with non-normally distributed variables. Scatter dot plots with mean, standard deviation, and significance were constructed based on the results of the independent samples t-test and the Mann–Whitney U test for CBMD, and MCW values between the different breathing patterns within the same age groups and different age groups (Figs. 6, and 7).

When comparing the CBMD and MCW values between the mouth and nasal breathers within the same age group, significant differences were found as follows: in the 7–9 years age group, MSPogCBMD、MSPogMCW、FMA (*P* = 0.028, 0.001,0.0001) showed significant differences; while in the 10–12 years age group, MSPogCBMD, MSMeCBMD, MSPogMCW, FMA and MSMeMCW (*P* = 0.003, 0.001, 0.001, 0.001,0.001) were differed significantly. The CBMD and MCW values were higher, and FMA values were lower in nasal breathers compared to mouth breather individuals.

When comparing the differences in CBMD and MCW between the different age groups within the same respiratory pattern, the following were observed: the sites that showed significant differences in nasal breather: MSPogCBMD, MSMeCBMD, MSGeCBMD, MSPogMCW, MSMeMCW, MSGeMCW, IMFMCW, B6MCW, L6MCW, I6MCW 、FMA、SNB(*P* = 0.001, 0.004, 0.012, 0.002, 0.001, 0.025, 0.001, 0.006, 0.001, 0.005, 0.008, 0.038). While in the mouth breather with different age groups, MSPogCBMD, MSMeCBMD, MSPogMCW, IMFMCW, B6MCW, I6MCW (*P* = 0.001, 0.032, 0.007, 0.001, 0.009, 0.011) showed statistically significant differences. In the 10–12 age group, CBMD, MCW, and SNB values were higher, and FMA was lower than that found in the 7–9 age group. The number of significantly different research sites in terms of CBMD and MCW was more in the nasal breathers group (10 sites) compared to the mouth breathers group (6 sites) (Table 2).

**Table 2:** Comparison of mandibular CBMD and MCW in 7–9, and 10–12 years old groups with different respiratory patterns

**Age**	**7-9Y**				**10-12Y**				**7-9Y VS 10-12Y**		**7-9Y VS 10-12Y**	
**RP**	**NB** **M (IQR)**	**MB** **M (IQR)**	**z/t**	***P***	**NB** **M (IQR)**	**MB** **M (IQR)**	**z/t**	***P***	**NB**		**MB**	
									**z/t**	**p**	**z/t**	***P***
**MSPogCBMD**^**b**^	1091.50(545.00)	999.50(315.76)	-2.19	0.028*	1602.50(538.00)	1387.00(487.00)	-2.958	0.003**	-3.401	0.001**	-3.413	0.001**
**MSMeCBMD**^**a**^	1170.70(± 401.75)	1005.18(± 343.83)	1.78	0.081	1471.77(± 358.25)	1133.94(± 351.50)	4.23	0.000***	-2.898	0.004**	-2.149	0.032*
**MSGeCBMD**^**b**^	937.50(497.25)	919.50(349.92)	-0.12	0.904	1122(291.415)	1057.50(282.25)	-1.345	0.179	-2.513	0.012*	-1.418	0.156
**IMFCBMD**^**b**^	1123.50(524.50)	1106.50(427.61)	-0.01	0.989	1269.50(363.16)	1241.50(321.50)	-0.859	0.39	-1.819	0.069	-0.577	0.564
**L6CBMD**^**b**^	670.00(144.63)	736.00(257.33)	-0.67	0.505	654.75(177.73)	688.50(316.25)	-0.592	0.554	-0.325	0.745	-0.449	0.653
**B6CBMD**^**b**^	645.50(412.75)	675.00(313.64)	-0.46	0.643	739.00(278.13)	687.50(277.75)	-1.113	0.266	-0.798	0.425	-0.154	0.878
**I6CBMD**^**a**^	1022.23(± 402.69)	1004.06(± 349.32)	0.19	0.847	1134.30(± 335.33)	1054.78(± 351.00)	1..006	0.32	-1.264	0.206	-0.93	0.352
**MSpogMCW**^**b**^	2.50(0.80)	1.90(0.50)	-3.33	0.001**	3.05(0.78)	2.25(0.60)	-4.241	0.000***	-3.095	0.002**	-2.696	0.007**
**MSmeMCW**^**b**^	2.35(0.90)	2.10(0.42)	-1.70	0.09	3.05(0.48)	2.30(0.60)	-4.626	0.000***	-3.866	0.000***	-1.832	0.067
**MSGeMCW**^**b**^	2.15(1.00)	2.45(0.45)	-1.24	0.214	2.80(0.68)	2.55(0.88)	-1.171	0.242	-2.239	0.025*	-0.322	0.748
**IMFMCW**^**b**^	2.20(0.48)	2.33(0.44)	-0.65	0.518	3.00(0.74)	2.73(0.95)	-0.55	0.583	-4.041	0.000***	-3.551	0.000***
**B6MCW**^**b**^	2.30(0.60)	2.33(0.45)	-0.29	0.772	2.65(0.60)	2.65(0.82)	-0.233	0.816	-2.761	0.006**	-2.626	0.009**
**L6MCW**^**a**^	1.83(± 0.363)	2.01(± 0.399)	-1.901	0.062	2.26(± 0.455)	2.16(± 0.64)	0.93	0.36	-3.507	0.000***	-1.587	0.112
**I6MCW**^**b**^	2.45(0.77)	2.25(0.431)	-1.145	0.252	2.73(0.642)	2.58(0.94)	-1.184	0.236	-2.819	0.005**	-2.556	0.011*
**FMA**^**b**^	28(4.38)	33(4.25)	-4.325	0.000***	26.12(2.0)	34(5.2)	-6.23	0.000***	-2.649	0.008 **	-0.709	0.479
**SNA**^**b**^	79.55(4.38)	80.9(5.22)	-1.433	0.152	81.30(4.25)	82.10(5.2)	-1.021	0.307	-1.878	0.06	-1.623	0.104
**SNB**^**a**^	75.33 (± 3.34)	74.7529(± 3.46)	0.174	0.678	77.05 (± 2.90)	76.24 (± 3.43)	0.809	0.372	-2.125	0.038*	-1.759	0.083
**ANB**^**a**^	4.2167 (± 1.83)	5.70(± 1.80)	0.013	0.91	4.04 (± 1.99)	5.56 (± 1.80)	1.525	0.222	0.351	0.727	0.310	0.757

^a^ Independent sample t-test

^b^ Mann–Whitney U test

^***^p < 0.001, **p < 0.01, *p < 0.05

The regression equation was formulated as logit(Y) = -21.537 + 0.002*X1-1.438*X2 + 0.723*X3 + 0.27*X4 (Y: Breathing pattern Nasal breathing or Mouth breathing; X1:MSpogCBMD; X2:MSpogMCW; X3:FMA: X4:ANB) MSPogMCW was the most infuencing factor, followed by FMA, ANB, and MSPogCBMD (*P* value: 0.026, 0.0001, 0.105 and 0.041 respectively); considering that the negative β value indicated decreased likelihood of falling into MB group, while the positive β value indicated increased likelihood of falling into NB group (Table 3).

**Table 3:** Binary logistic regression analysis

	β	SE	Wald	*P*	OR	95% CI for odds ratio	
						Upper	Lower
FMA	0.723	0.138	27.241	0.000	2.06	1.57	2.703
MSPogCBMD	0.002	0.001	4.157	0.041	1.002	1	1.003
MSpogMCW	-1.438	0.646	4.954	0.026	0.237	0.067	0.842
ANB	0.27	0.167	2.627	0.105	1.31	0.945	1.817
Constant	-21.537	4.809	20.057	0.000	0.000	-	-

**Fig. 8:**
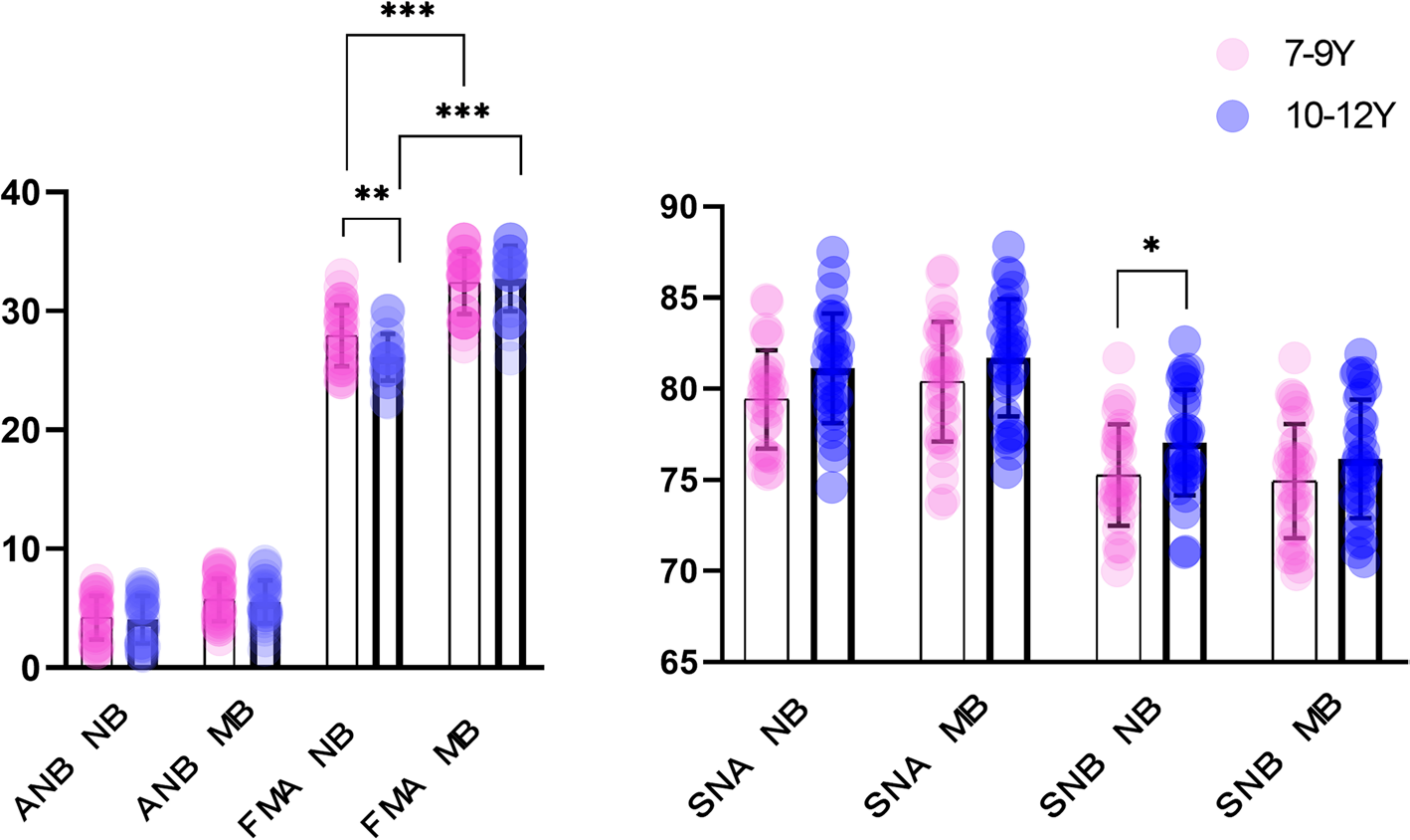
Vertical and sagittal difference analysis of different age groups with the same and different breathing patterns

There is a significant upward trend in the vertical angle of MB relative to the NB pattern in both age groups. The mandible of the 10–12 years old NB group was significantly increased in sagittal direction than that of MB group. NB patterns showed more average angular patterns at ages 10 to 12 and higher angular patterns at 7 to 9 years (Fig. 8).

**Fig. 9:**
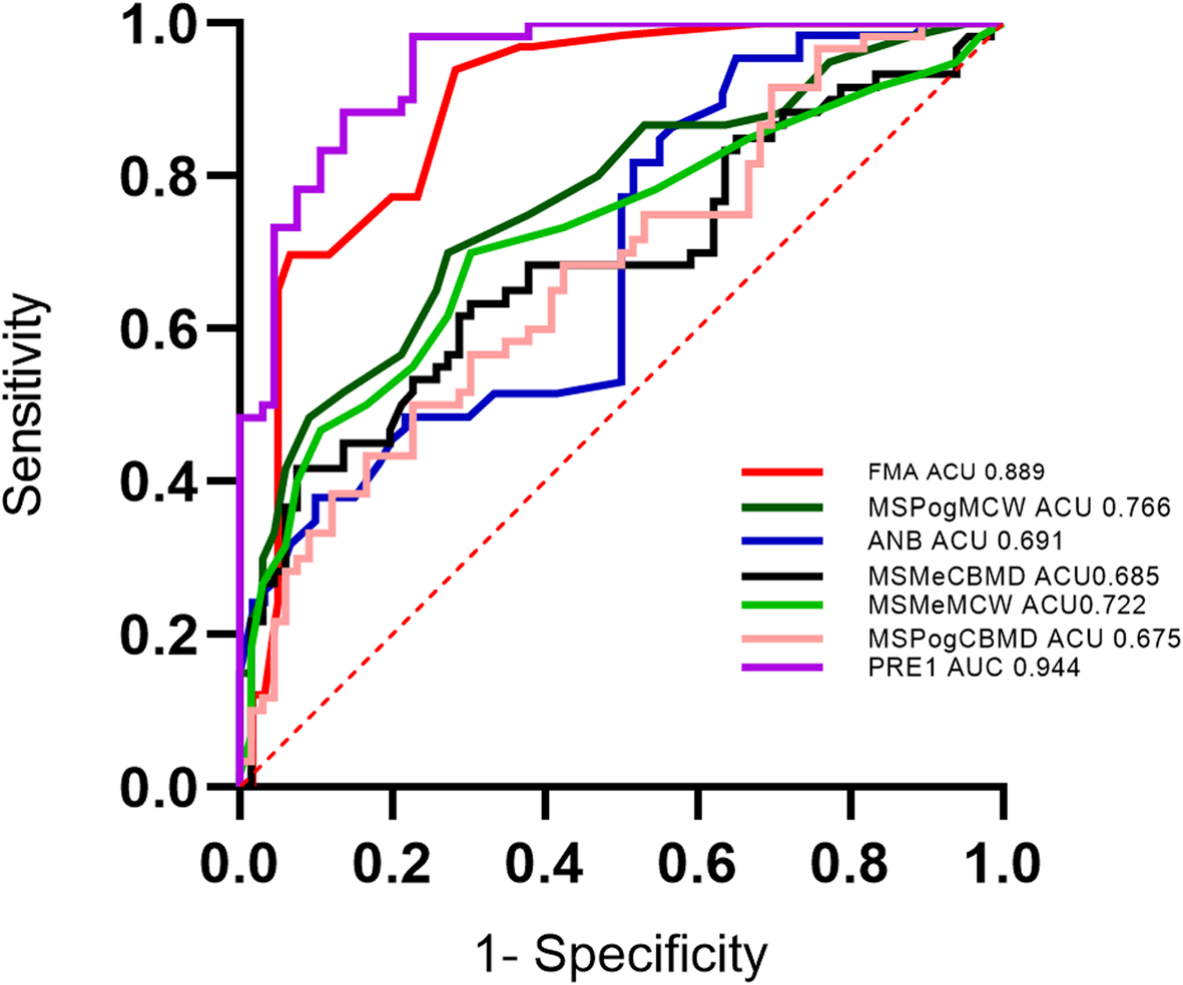
Diagnostic prediction of subject working characteristic (ROC) curve and respiratory pattern

ROC curve analysis showed that the area under the curve was PR1, FMA, MSpogMCW, MSMeMCW, ANB, MSMeCBMD, and MSPogCBMD from large to small (Table 4). The accuracy, sensitivity, and specificity of the combined diagnosis were significantly higher than those of ANB, FMA, MSPogCBMD, MSMeCBMD, MSPogMCW, and MSMeMCW (Fig. 9).

**Table 4:** Statistical analysis of receiver operating characteristic curve

	AUC	*P* value	Sensitivity	Specificity	cutoff scores	95% CI for odds ratio	
						Lower	Upper
MSPogCBMD	0.675	.001	0.773	0.500	1440.0	.582	.768
MSMeCBMD	0.685	.000	0.910	0.420	1413.5	.591	.780
MSpogMCW	0.766	.000	0.727	0.700	2.35	.683	.849
MSmeMCW	0.722	.000	0.700	0.700	2.35	.631	.813
ANB	0.691	0.000	0.955	0.953	3.15	0.599	0.783
FMA	0.889	0.000	0.697	0.977	31.5	0.872	0.962
PR1	0.944	0.000	0.773	0.964	0.765	0.785	0.927

^***^p < 0.001, **p < 0.01, *p < 0.05

## Discussion

In the selection of measurement sites during the investigation, we referred to a study that used CBCT to assess mandibular symphysis bone density in different skeletal patterns for anterior mandibular bone evaluation [15]. For the middle area of the mandible, we have chosen the cortical bone below the mental foramen based on previous literatures, which demonstrated robust sensitivity (0.602; 95% confidence interval [CI], 0.398–0.775) and specificity (0.708; 95% CI, 0.568–0.817) [16]. The population in our study mainly consisted of individuals aged 7–12 years with mixed dentition. Considering Han et al. research [17], we selected the cortical bone corresponding to the midpoint of the roots of the first molar for cortical bone evaluation in the posterior segment of the mandible.

This study has unveiled a significant correlation between children’s respiratory patterns and cortical bone mineral density (CBMD) and mandibular cortical width (MCW) at different age groups. Among 7–9-year-olds, CBMDPog exhibited a substantial increase of approximately 9.2%, and MCW Pog demonstrated an even more pronounced augmentation, with values approximately 31.6% higher in nasal breathers compared to mouth breathers, highlighting the influence of respiratory patterns on craniofacial development at the anterior chin point (Pog). Similarly, notable differences were observed in the 10–12-year-old group in CBMD and MCW values. CBMD at Pog showed a significant increase of about 15.5% in nasal breathers, while MCW exhibited a substantial rise of approximately 35%. At the Menton point, CBMD displayed a significant 30% increase, and MCW demonstrated an approximately 32.6% rise in nasal breathers compared to their mouth-breathing counterparts. These findings underscore the importance of nasal breathing in fostering improved CBMD and MCW at specific mandibular points in different age groups, highlighting the potential impact of respiratory patterns on craniofacial development.

The analysis suggests this may be related to intermittent hypoxia (IH) caused by mouth breathing which can affect mandibular bone development. IH can lead to sleep disorders that cause changes in hormone levels and disruptions in melatonin secretion, a known regulator of bone mass. The sympathetic nervous system (SNS) coordinates various physiological functions, including the sleep/wake cycle. Sleep disorders can lead to excessive SNS activity, resulting in decreased bone mass. Hong et al. have demonstrated that activation of β2-adrenergic receptors during growth may induce delayed mandibular bone growth in rats with IH [18]. Swanson et al. have also described the importance of the circadian rhythm of bone remodeling. Sleep disorders, decreased sleep quality, nocturnal hypoxia, inflammation, and other factors can disrupt this rhythm and/or affect bone metabolism through other mechanisms, making individuals more prone to low bone mass and fractures [19].

The relationship between malocclusion and breathing patterns is an interrelated phenomenon. Malocclusion can contribute to improper breathing patterns, and conversely, improper breathing patterns can exacerbate malocclusion [20]. Our study’s findings provide critical insights into the latter aspect, showing how nasal breathing may positively influence CBMD and MCW at specific mandibular points in different age groups. This implies that interventions targeting improved breathing patterns could potentially have a positive impact on craniofacial development.

Malocclusions are multifaceted conditions that affect dental and craniofacial structures and can also participate in temporomandibular disorders (TMD) development. Boening et al. [21] sheds light on the relationship between malocclusions and TMD and reports that malocclusions can lead to dysfunctional occlusal relationships, contributing to TMD symptoms, such as jaw pain, clicking, or limited mouth opening. Therefore, the alignment of dental arches, influenced by malocclusion, can either alleviate or exacerbate TMD symptoms in individuals with different breathing patterns.

Animal experiments have also demonstrated a correlation between mouth breathing, delayed mandibular bone metabolism, and low mandibular bone density. Raff et al. and Song et al. reported that bone density decreased in rats exposed to a low oxygen environment [22, 23]. Kim et al. [24] and Wang et al. [25] have established a model of unilateral nasal obstruction in SD rats and used micro-computed tomography (m-CT), stereomicroscopy, and immunohistochemistry methods for investigation. The results showed that nasal obstruction led to overall growth and organ development retardation due to mouth breathing (chronic hypoxia), decreased mandibular bone density (BMD) and bone volume/total volume ratio (BV/TV), increased osteoclast differentiation caused by activation of inflammatory reactions, leading to destructive changes in the alveolar bone. Additionally, Hosomichi et al. found that intermittent hypoxia can cause delayed mandibular bone growth in growing rats [26–28].

In addition to hypoxia, abnormal muscle movements accompanying oral breathing patterns are associated with adaptive jaw shape and density changes. According to the Moss theory, skeletal muscle contractions are typical loading events for the functional matrix of bone [29]. This process extends from skeletal muscle contractions to the regulation of the bone cell genome. The Functional Matrix Hypothesis (FMH) of intracellular mechano-sensing, mechano-transduction, and intercellular communication capabilities provided an explanatory chain, suggesting that bone adaptation is a tightly coordinated process between the skeleton and skeletal muscles. Zhao et al. conducted a study that demonstrated that breathing patterns could alter the electromyographic activity of craniofacial skeletal muscles, leading to changes in neuromuscular activity and affecting muscle development and skeletal remodeling [30].

Our study found that the nasal breathing pattern in the 10–12-year-old group was associated with a higher prevalence of a balanced facial profile. In comparison, the 7–9-year-old group had a higher prevalence of high-angle facial profiles. This may be related to dietary habits in children, where younger children tend to consume softer and more easily digestible food [28]. Children with mouth breathing may have a sagittal deformity of the maxilla and mandible manifested by maxillary protrusion and mandibular retrusion [29], which is also confirmed in this study.

Our research highlights the critical relationship between respiratory patterns and craniofacial development. Acknowledging that the upper airway volume plays a pivotal role in this complex interplay is important. Dastan et al. [31] has investigated the correlations between upper airway volume and various craniofacial parameters, including the position of the hyoid bone, palatal depth, nasal septum deviation, and concha bullosa, across different types of malocclusion. These findings emphasize the far-reaching consequences of airway anatomy on facial morphology and underscore the need for a comprehensive approach to diagnosing and addressing airway issues in individuals with malocclusion.

Furthermore, we have proposed a method that combines sagittal and vertical bone profiles with cortical bone width and density to provide an objective evaluation index for the initial screening and classification of mouth and nasal breathing patterns in children. Experimental results showed that under the same conditions, the accuracy rate of breathing pattern inference of the binary logistic correlation model was 85.7%, surpassing the single data source method. In addition to validating the existing symptoms in children with mouth breathing patterns, this study further explored other factors related to craniofacial manifestations, providing secondary prevention evidence for the early detection, diagnosis, and treatment of oral breathing system disorders.

Bone metabolism is a complex process influenced by various factors such as dietary habits, the severity of mouth breathing disorders, and the duration of the condition. Due to the limited sample size, the study did not include children with skeletal Class III malocclusion. Future studies should increase sample sizes and employ rigorous experimental designs and prospective research approaches to further investigate these relationships. Additionally, it would be beneficial to establish reference datasets representing average jawbone quality for different regions, ages, genders, and ethnicities. Comparing bone tissues from stable craniofacial or body structure regions as control variables could also help validate the relationship between low bone density, skeletal patterns, and breathing patterns.

## Conclusion

The oral respiratory pattern may lead to the decreased trend of mandibular CBMD and MCW in children aged 7–12 years, and the Pog and Me sites in the lower anterior mandible are more significantly affected. The combined diagnosis with bone facial pattern variables provides a certain basis for the early warning of oral respiratory pattern.

## Data Availability

Data used and/or analyzed during the current study are available from the corresponding author upon reasonable request.

## Abbreviations

MB: Mouth breathing
NB: Nasal breathing
CBCT: Cone-beam computed tomography
3D: Three dimensional
DICOM: Digital imaging and communication
CBMD: Cortical Bone Mineral Density
MCW: Mandibular Cortical Width
